# The miR-1843a-3p/*Mef2c/Egr1* Axis Is Associated with Prenatal Gamma Radiation-Induced Deficits in Adult Hippocampal Neurogenesis and Behaviour

**DOI:** 10.3390/cells15100912

**Published:** 2026-05-15

**Authors:** Yunwei Shi, Hong Wang, Nur Salihah Lau, Amanda Tan Ying Xin, Caiping Wang, Feng Ru Tang

**Affiliations:** 1 Jiangsu Key Laboratory of Tissue Engineering and Neuroregeneration, Key Laboratory of Neuroregeneration of Ministry of Education, Co-Innovation Center of Neuroregeneration, NMPA Key Laboratory for Research and Evaluation of Tissue Engineering Technology Products, Nantong University, Nantong 226001, China; 2 Radiation Physiology Laboratory, Singapore Nuclear Research and Safety Institute, National University of Singapore, 16 Prince George’s Park, #03-03, Singapore 118415, Singapore; snrwh@nus.edu.sg (H.W.);

**Keywords:** prenatal gamma irradiation, neurogenesis, behavioural abnormalities, miR-1843a-3p, Mef2c, Egr1

## Abstract

**Highlights:**

**What are the main findings?**

- Firstly, chronic prenatal gamma irradiation in mice increases depression-like behaviors in adult offspring, attributing to reduced adult hippocampal neurogenesis characterized by decreased DCX-positive newborn neurons and NeuN-positive mature neurons in the dentate gyrus.
- Secondly, the miR-1843a-3p/Mef2c/Egr1 axis is identified as a novel regulatory pathway mediating the long-term neurodevelopmental consequences of prenatal irradiation exposure, with miR-1843a-3p directly targeting Mef2c.

**What are the implications of the main findings?**

- Firstly, hippocampal injury induced by chronic prenatal gamma exposure in mice persists into adulthood.
- Secondly, the signaling pathway mediating neurodevelopmental deficits induced by radiation-induced suggests potential neuroprotective targets.

**Abstract:**

Prenatal exposure to ionizing radiation is a known risk factor for neurodevelopmental deficits; however, the molecular mechanisms linking chronic embryonic insult to abnormal brain development remain poorly understood. This study investigated the long-term consequences of chronic prenatal gamma irradiation throughout gestation in C57BL/6 mice. Behavioural analysis of adult offspring revealed a specific increase in depression-like behaviours, with no significant alterations in anxiety or general exploratory activity. Immunohistochemical assessment demonstrated a significant reduction in adult hippocampal neurogenesis, marked by decreased doublecortin (DCX)-positive newborn neurons in the subgranular zone and fewer NeuN-positive mature neurons in the dentate gyrus hilus. Integrated RNA-seq, qPCR, and Western blot analyses implicated the upregulation of the Mef2c/Egr1 signalling pathway in this neurogenic deficit. Furthermore, miRNA sequencing identified a pronounced decrease in miR-1843a-3p, which was subsequently validated to directly target Mef2c. Collectively, these findings suggest that prenatal gamma irradiation disrupts neurogenic processes and adult brain function, leading to specific behavioral abnormalities. This long-term impairment is associated with, and may be at least partially mediated by, dysregulation of the miR-1843a-3p/Mef2c/Egr1 pathway.

## 1. Introduction

The prenatal period represents a critical window of heightened sensitivity to environmental teratogens. Ionizing radiation is a potent example, with high-dose exposures known to cause severe structural and functional impairments [1]. While the consequences of acute, high-dose exposures are well-documented, the long-term outcomes of prolonged gestational exposure are less understood, particularly under differing dose-rate paradigms.

Recent work has established that continuous, low-dose-rate (100 mGy/day) prenatal exposure, cumulatively reaching a high total dose (1.8 Gy), can induce significant molecular alterations in mRNA and miRNA profiles in the adult hippocampus without concomitant cellular changes in the dentate gyrus or overt neuropsychiatric deficits [2]. This dissociation between molecular perturbation and functional outcome highlights the complexity of radiation-induced neuropathology. In contrast, the impact of a high-dose-rate prenatal exposure, designed to deliver a similar cumulative high dose (~5 Gy) as an acute neonatal exposure in our previous studies [1], remains entirely unexplored. This specific high-dose-rate gestational model is critically relevant for understanding scenarios such as nuclear accidents, where a high-intensity fetal exposure may occur before protective evacuation.

The molecular mechanisms that translate such developmental insults into latent risk for adult brain dysfunction are poorly defined. Identifying the key dysregulated pathways is therefore essential for developing predictive biomarkers and targeted neuroprotective strategies. Emerging evidence points to the disruption of activity-dependent transcriptional networks as a central mechanism in the long-term programming of neural circuits following developmental insults [3]. Prominent candidates within these networks include myocyte enhancer factor 2C (Mef2c) and early growth response 1 (Egr1). Mef2c is vital for synaptic development, plasticity, and neuronal survival [4], while Egr1 is an immediate-early gene critical for memory consolidation and adaptation [5,6]. Both factors are sensitive to environmental stressors and engage in a coordinated regulatory dialogue; Egr1 can modulate Mef2c expression, and Mef2c activity can influence Egr1 regulatory pathways, positioning them within a shared vulnerability network [7,8].

Therefore, this study tests the hypothesis that chronic, high-dose-rate prenatal gamma irradiation induces a distinct profile of hippocampal damage and behavioral dysfunction in adult C57BL/6 mice compared to the sequelae of acute neonatal exposure at an equivalent total dose. We further propose that this outcome is mechanistically driven by the specific disruption of the Mef2c/Egr1 regulatory axis. By elucidating this pathway, our work aims to define novel molecular links between the dynamics of prenatal radiation exposure and the pathogenesis of adult-onset neurological deficits.

## 2. Materials and Methods

### 2.1. Animal Experiments

C57BL/6 mice were supplied by InVivos Pte. Ltd. (Singapore) and housed in the Comparative Medicine Facility in the National University of Singapore. Pregnant dams were randomly assigned to control or irradiation groups. In the experiment group, mice were irradiated with a dose-rate 12 mGy/h for 18 days (radiation exposure time 23.5 h/day) (Hopewell G10-1-12 γ-Irradiator, Hopewell Designs, Inc., Alpharetta, GA, USA) with an accumulative dose of ~5 Gy. Offspring in the control and experimental groups were fed in the same environment for 6 months. For behavioral testing, mice were coded by an investigator not involved in data collection, and all behavioral analyses, including elevated plus maze, light dark box, open field, novel object recognition, tail suspension test, and forced swim test, were performed blind to group allocation. Sample sizes (*n* = 10 per group for behavioral tests) were determined based on power analysis from our previous studies [1,2] and are indicated in each figure legend.

The results were recorded and analyzed by ANY-maze software version 7.10 (ANY-maze, Wood Dale, IL, USA). Only male offspring were used in this study to avoid potential confounding effects of the estrous cycle on behavioral outcomes.

All these animal handling experiments were approved by the Institutional Animal Care and Use Committee (IACUC), National University of Singapore (IACUC number: R20-103).

### 2.2. Behavioral Assessments

#### 2.2.1. Elevated Plus Maze

The apparatus was raised 50 cm above the floor. It comprised two open arms (5 × 30 cm), two closed arms (same dimensions), and a central square (5 × 5 cm). A mouse was introduced into one closed arm, after which a 10 min recording session began. Parameters measured included travel distance and duration of stays in open arms, closed arms, and the center zone.

#### 2.2.2. Light Dark Box

A 50 × 50 cm enclosure was split into two equal 50 × 25 cm compartments: one brightly lit (light zone) and the other darkened by a cover (dark zone). Each mouse started in the light side, with behavior tracked for 10 min. The system logged time spent and distance moved only within the light zone.

#### 2.2.3. Open Field (Locomotor) Test

Testing was conducted in a non-transparent, empty square arena (50 × 50 cm). Software divided the floor into three regions: center, corners, and periphery. The animal began in the center and explored freely for 30 min. Outcome measures were time spent and distance travelled within each region.

#### 2.2.4. Novel Object Recognition Test

The procedure spanned four days: two habituation sessions, one training day, and one test day. On habituation days, mice roamed an empty 50 × 25 cm box for 10 min. During training, two identical objects (square rubber waffles) were placed on opposite sides. Twenty four hours later, one waffle was swapped for a novel circular plastic bowl. Before each animal, the arena and objects were cleaned sequentially with Clidox and ethanol. The ANY maze software defined a 3 cm radius investigation zone around each object, automatically measuring the time the mouse spent inside those zones.

#### 2.2.5. Tail Suspension Test (TST)

Each mouse was taped by the tail to a hook and suspended for 6 min. Immobility, defined as the absence of any limb movement for at least 2 s was recorded. Longer immobility indicated greater depression-like behavior.

#### 2.2.6. Forced Swim Test (FST)

A cylindrical tank (20 cm diameter) held water at 24–26 °C. Mice were placed inside and allowed to swim freely for 8 min. The percentage of time spent immobile (floating with only the head above water and no limb motion for ≥ 2 s) served as the index of depression-like behavior.

### 2.3. Animal Sample Collection

Mice were euthanized by CO_2_. The brain was removed and cut into the left and right hemispheres. The right-side hemisphere was fixed with 4% paraformaldehyde in 0.1 M PBS (pH = 7.4). The left side hippocampus and cortex were dissected and frozen in liquid nitrogen. One day after fixation, the right brain was transferred to 30% sucrose.

### 2.4. Immunohistochemical Staining

Immunohistochemical staining was performed according to our previous studies [1,2]. Sagittal brain sections were cut at 40 μm, treated with 3% H_2_O_2_, and blocked by 4% normal goat serum at room temperature. These free-floating sections were incubated with antibodies against doublecortin (DCX, 1:500; Santa Cruz Biotechnology Inc., Santa Cruz, CA, USA), NeuN (1:500; Gene Tex, Hsinchu City, Taiwan), MEF2C (1:400; Cell Signaling Technology, Danvers, MA, USA), or EGR1 (1:200; Thermo Fisher Scientific, Waltham, MA, USA) overnight. The sections were then washed and incubated with respective secondary antibodies, followed by avidin–biotin complex (ABC) reagent (Vector Laboratories Inc., Burlingame, CA, USA). After reaction in DAB Peroxidase Substrate (Vector Laboratories Inc., Burlingame, CA, USA), the sections were washed, mounted, counterstained and covered. The slides were examined and photographed under microscopy (Leica Microsystems GmbH, Wetzlar, Germany). The Stereologer System (Stereology Resource Center, Biosciences Inc., Tampa, FL, USA) was used to unbiasedly analyze the number of DCX and DCX immunopositive cells.

### 2.5. Total RNA Isolation from Murine Hippocampus

Total RNA was extracted from the hippocampus of both control and prenatally irradiated animals. The miRNeasy Mini Kit (Qiagen, Hilden, Germany) was employed, and all steps followed the manufacturer’s specifications as previously described [2]. In brief, each hippocampal sample was homogenized with 140 µL of chloroform. After centrifugation, the upper aqueous phase containing RNA was collected, combined with absolute ethanol, and applied to an RNeasy Mini spin column. RNA was eluted from the column membrane by centrifugation and redissolved in RNase free water. RNA concentration and integrity were verified using a Nanodrop spectrophotometer and a Bioanalyzer system (Agilent Technologies, Santa Clara, CA, USA) before being used for miRNA sequencing (miRSeq) and qRT PCR.

### 2.6. Systematic mRNA and miRNA Sequencing Analysis

mRNA and miRNA sequencing of hippocampal samples was performed using protocols reported earlier [2]. For mRNA sequencing, RNA samples were first denatured, enriched, and fragmented. Following the synthesis of the first and second strand cDNA, the double stranded product was end repaired. A single adenine nucleotide was added to the 3′ ends of the blunt fragments, after which adaptors were ligated, and PCR amplification was carried out. Finally, DNA nanoballs containing multiple copies of DNA were generated and sequenced.

For miRSeq, RNA samples were ligated sequentially to 3′ and 5′ adapters. RT PCR products were purified by PAGE gel electrophoresis. DNA nanoballs were then produced and sequenced using Probe Anchor Synthesis (cPAS). All sequencing data were analyzed on the DNBSEQ platform (BGI Genomics, Shenzhen, China). Differentially expressed genes and miRNAs were identified using DESeq2 with Benjamini–Hochberg false discovery rate (FDR) correction for multiple comparisons (FDR < 0.05 and |log2FC| > 0.585). This analysis identified 191 mRNAs and 43 miRNAs as differentially expressed in the irradiated hippocampus.

### 2.7. RT-qPCR Analyses

cDNA was synthesized using a Maxima First Strand cDNA Synthesis kit. A total of 2 μg RNA was mixed with 2 μL Maxima Enzyme Mix, and the 4 μL reaction mix (5×) contains the remaining reaction components: reaction buffer, dNTPs, oligo(dT)18, and random hexamer primers, for a total of 20 μL reaction volume. cDNA synthesis reaction was performed at 25 °C for 10 min, 50 °C for 30 min, and 85 °C for 5 min. Real-time PCR was performed in triplicate in the Applied Biosystems™ QuantStudio 6 Flex Real-Time PCR System (Thermo Fisher Scientific, Singapore). A final 20 μL volume included 2 μL cDNA; 10 μL Maxima SYBR Green/ROX qPCR Master Mix (2×) containing Maxima Hot Start Taq DNA Polymerase, SYBR Green I, ROX passive reference dye, and dNTPs (also dUTP) in an optimized PCR buffer; and 1 μM each of the respective forward and reverse primers. Reactions were performed at 50 °C for 2 min and 95 °C for 10 min, 40 cycles of denaturation at 95 °C for 15 s, and annealing and extension at 60 °C for 30 s and 72 °C for 30 s. The amount of the target gene was normalized to GAPDH and was calculated by the 2^−ΔΔCT^ method. Results were expressed as the fold change to the control group. The primers used for real-time PCR are listed in Table 1.

### 2.8. Western Blot

The mouse hippocampus was weighed and added to 20× CelLytic^MT^ Mammalian Tissue Lysis/Extraction Reagent (Sigma-Aldrich Corporation, St. Louis, MO, USA) containing 100× Halt™ Protease and Phosphatase Inhibitor Cocktail (Thermo Fisher Scientific, Waltham, MA, USA). The tissue was homogenized in an ice bath. The lysate was centrifuged at 15,000× *g* for 15 min, and the supernatant was transferred into a pre-chilled tube. The protein concentration was determined with Pierce™ BCA Protein Assay Kit (Thermo Fisher Scientific, Waltham, MA, USA). All the protein samples were diluted to the same concentration with RIPA solution and were then added 4× loading buffer for later use. Protein lysates were separated by 8–12% SDS-PAGE gels and then transferred to a nitrocellulose membrane. The membrane was blocked by Blotting One solution (Nacalai Tesque Inc., Kyoto, Japan), incubated with the respective primary antibodies overnight at 4 °C followed by HRP-conjugated secondary antibodies at room temperature for 1 h. The WesternBright Sirius Chemiluminescent Detection Kit (Advansta Inc., San Jose, CA, USA) was used to detect immunoreactive proteins. Membranes were then visualized and quantified using the Bio-Rad Gel Doc system. Band strip gray values were measured by ImageJ 1.53a software and normalized by the respective loading control β-actin. The fold change relative to the control was calculated.

### 2.9. Predication of Mef2c Upstream miRNA Targets and Luciferase Reporter Assay

Online database TargetScan was utilized to analyze and predict the potential interactions of miRNAs with *Mef2c*. mmu-miR-34b-3p, mmu-miR-34c-3p, mmu-miR-1264-5p, and mmu-miR-1843a-3p were selected for further validating their direct interaction with *Mef2c* by the luciferase reporter assay.

RT-PCR amplified the mouse *Mef2c* 3′UTR region containing the potential binding sequence of miRNAs. The primer sequences were listed in Table 2. The amplified fragments were directionally cloned into the XhoI and NotI unique restriction enzyme sites of the psiCHECK-2 plasmid, which are downstream of the Renilla luciferase gene. Transfection efficiency was normalized using firefly luciferase. The seed regions of miRNAs were mutated using the Phusion Site-Directed Mutagenesis Kit (Thermo Fisher Scientific, Waltham, MA, USA) with primer sequences listed in Table 2.

HEK293T cells were co-transfected with psiCHECK-2 constructed with 3′UTR binding sites of these miRNAs and their mimics or scrambled mimic controls respectively, using Lipofectamine 3000 transfection reagent (Thermo Fisher Scientific, Waltham, MA, USA) according to the manufacturer’s protocol. The Dual-Luciferase^®^ Reporter Assay System (Promega Corporation, Madison, WI, USA) was used to measure luciferase and Renilla signals 48 h after transfection.

### 2.10. Statistical Analysis

Student’s *t*-test was used to compare the body weight, behavioural changes, immunostained cells, and mRNA expression by qRT-PCR between control and irradiated mice. *p* < 0.05 was considered statistically significant. mRNA and miRNA analyses were based on the parameters |log2FC| > 0.585 and *p* < 0.05, which were considered as significantly differential expression of DEseq2.

## 3. Results

### 3.1. Prenatal Gamma Irradiation Induced Specific Behavioural Deficits in Adult Mice

**Figure 1:**
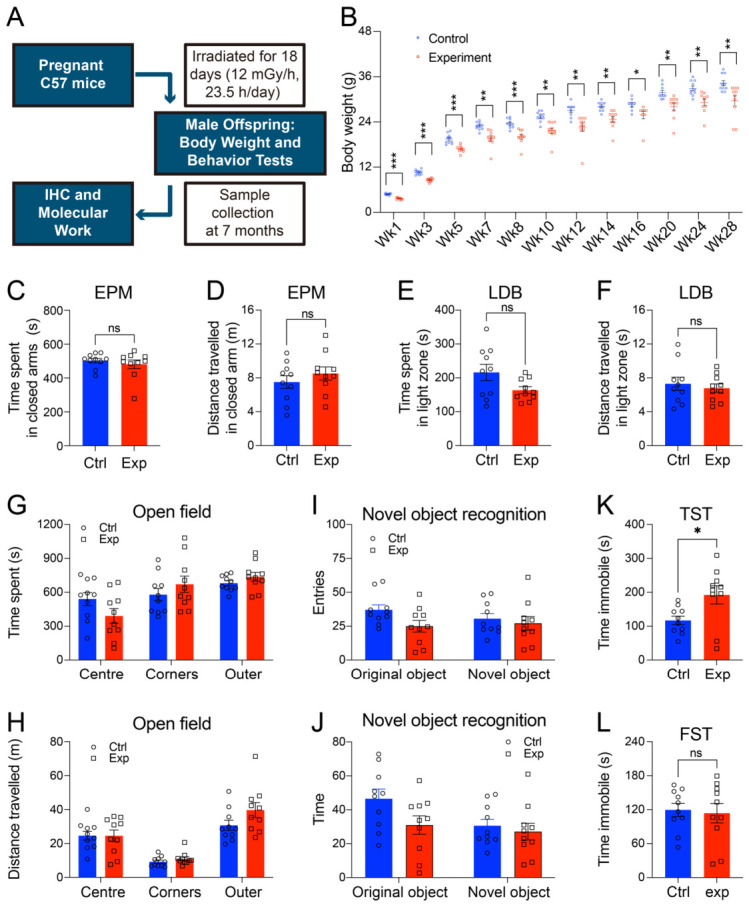
Prenatal irradiation-induced brain function impairment in mice. (**A**) Experimental workflow. (**B**) Body weight changes in control and prenatally irradiated C57BL/6 mice. Behavioural outcomes: elevated plus maze: time spent (**C**) and distance traveled (**D**) in the closed arms; light-dark box: time spent (**E**) and distance traveled (**F**) in the light zone; open field test: time spent (**G**) and distance travelled (**H**) in different areas; novel object recognition: entries (**I**) and time (**J**) in the original and novel object areas; tail suspension test: immobility time (**K**); forced swimming test: immobility time (**L**). Data are presented as mean ± SEM (*n* = 10 for each of the control and experimental groups). * *p* < 0.05, ** *p* < 0.01, *** *p* < 0.001. ns: not significant.

To assess the long-term physiological impact of prenatal irradiation, we monitored body weight in mice from postnatal week 1 to 28. A significant reduction in total body weight was observed in irradiated mice compared to controls (Figure 1B).

A comprehensive behavioural battery was employed to evaluate the functional consequences of prenatal exposure. Irradiated mice exhibited a pronounced depression-like phenotype in the tail suspension test indicted by a significant increase in immobility time (Figure 1K). However, irradiated animals did not show more time immobile in the forced swim test (Figure 1L). Literature also showed that the pathophysiology underlying TST and FST is different, and TST seems to be more sensitive to acute neurochemical changes than FST [9].

In contrast, performance in tests for anxiety and memory remained intact. No significant differences were observed between irradiated and control groups in the elevated plus maze (Figure 1C,D), light-dark box (Figure 1E,F), or open field test (Figure 1G,H). indicating unaffected anxiety-like behaviour and general locomotor exploration. Similarly, cognitive function, as assessed by the novel object recognition (Figure 1I,J), was not impaired.

These results demonstrate that chronic prenatal gamma irradiation induces a specific long-term phenotype in adult mice, characterized by reduced body weight and a selective increase in depression-like behaviours, without affecting anxiety levels, general activity, or hippocampal-dependent memory.

### 3.2. Prenatal Irradiation Disrupted Hippocampal Neurogenesis and Neuronal Maturation

**Figure 2:**
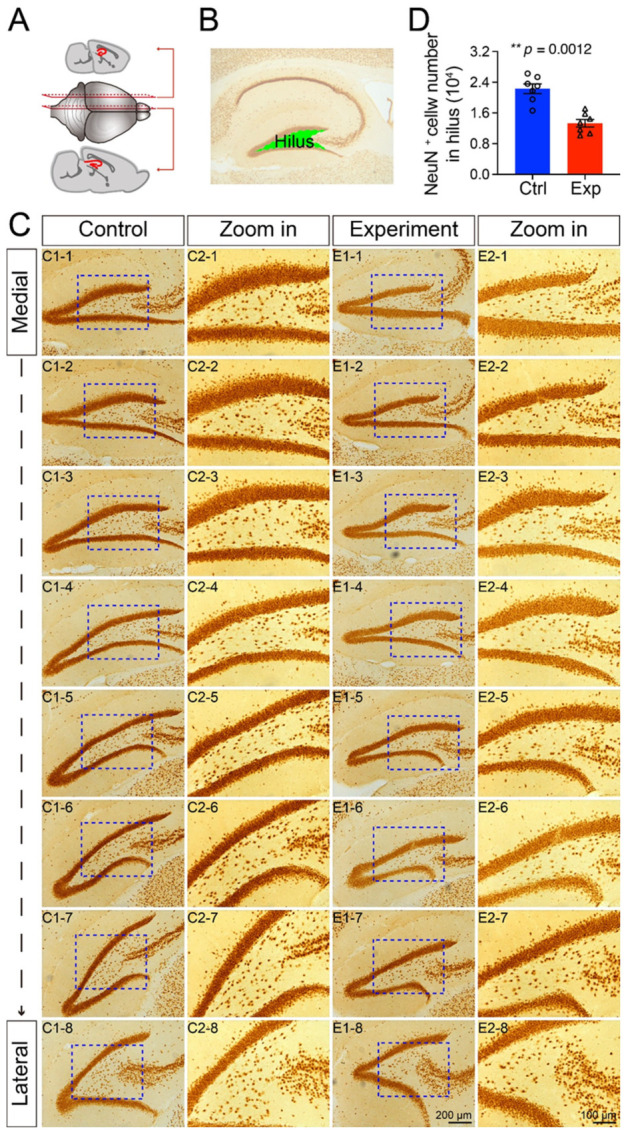
NeuN-immunopositive cells in the hippocampal hilus region. (**A**) Schematic representation of the division of hippocampal sagittal slices. (**B**) Anatomical localization of the hilus region within the hippocampus. (**C**) Representative immunohistochemical staining of NeuN showing medial-to-lateral sections. (**D**) Quantitative analysis of NeuN-immunopositive cell numbers in the hilus region of control and prenatally irradiated mice. All data are presented as mean ± SEM (*n* = 7 per group). ** *p* < 0.01 indicates a statistically significant difference between groups. Scale bar = 200 µm in E1-8 applies to C1-1 to C1-8, E1-1 to E1-7. Scale bar = 100 µm in E2-8 applies to C2-1 to C2-8, E2-1 to E2-7.

Immunohistochemical analysis revealed significant alterations in neuronal populations within the hippocampal dentate gyrus of irradiated mice. First, we observed a marked reduction in the number of NeuN-positive mature neurons within the hilus compared to controls (Figure 2C,D), indicating a specific deficit in this neuronal compartment.

**Figure 3:**
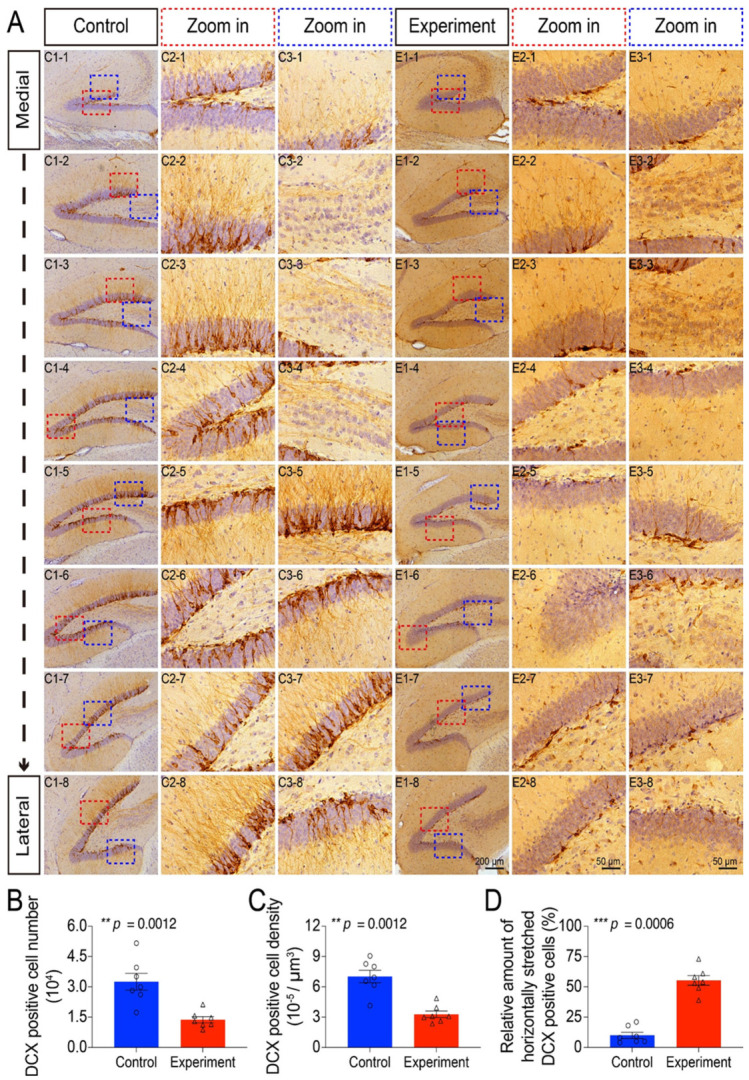
DCX-immunopositive cells in the granular layer of the dentate gyrus. (**A**) Representative immunohistochemical staining of DCX from medial to lateral sections. In irradiated mice, DCX-positive cells in the granular layer exhibited reduced cell number, cell aggregation, disordered process extension, and shortened processes. Quantification of DCX-immunopositive cell number (**B**), cell density (**C**), and horizontally oriented DCX-positive cells (**D**) in the granular layer of the dentate gyrus in control and prenatally irradiated mice. Data are presented as mean ± SEM (*n* = 7 per group). ** *p* < 0.01, *** *p* < 0.001. Scale bar = 200 µm in E1-8 applies to C1-1 to C1-8, E1-1 to E1-7. Scale bar = 50 µm in E2-8 applies to C2-1 to C2-8, E2-1 to E2-7. Scale bar = 50 µm in E3-8 applies to C3-1 to C3-8, E3-1 to E3-7.

We next assessed adult neurogenesis by examining doublecortin-positive (DCX^+^) newborn neurons. Prenatal irradiation resulted in a pronounced disruption of this population. Qualitative analysis revealed not only a reduction in the number of DCX^+^ cells but also severe morphological abnormalities, including disorganized dendritic processes, aberrant cellular clustering, and a decrease in mossy fibers connecting the hilus to the CA3 region (Figure 3A).

Quantification confirmed these observations. The total number and density of DCX^+^ cells were significantly diminished in irradiated animals (Figure 3B,C). Furthermore, there was a significant increase in the proportion of DCX^+^ cells with horizontally oriented processes (Figure 3D), suggesting a disruption in the normal radial migration and maturation trajectory of newborn neurons.

### 3.3. RNA-seq Analysis Identified the Dysregulation of Mef2c Transcriptional Network

**Figure 4:**
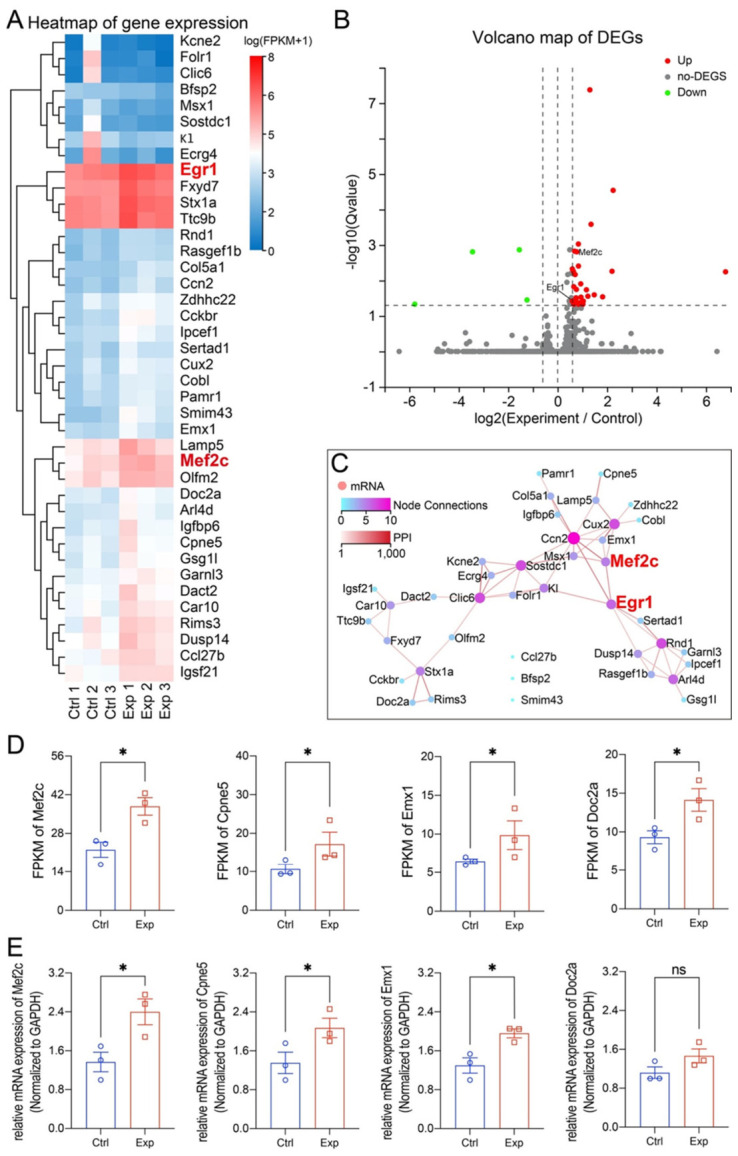
mRNA-seq analysis of hippocampal samples from control and irradiated mice. (**A**) Heatmap, (**B**) volcano plot, and (**C**) PPI network of the top 40 differentially expressed genes (DEGs; fold change ≥ 1.5, *p* < 0.05). (**D**) Comparison of FPKM values for selected DEGs, Mef2c, Cpne5, Emx1, and Doc2a, between control and irradiated groups. (**E**) qRT-PCR validation of expression changes for the selected genes. Data are shown as mean ± SEM; *n* = 3 per group; * *p* < 0.05. ns: not significant.

To elucidate the molecular mechanisms underlying the irradiation-induced neural and behavioural phenotypes, we performed RNA-seq on hippocampal tissue. Differential expression analysis identified 191 genes that were significantly dysregulated (*p* < 0.05; fold-change > 1.5) in irradiated mice compared to controls. A heatmap and volcano plot of the top 40 differentially expressed genes (DEGs) are shown in Figure 4A,B.

Strikingly, the transcription factor *Mef2c* was significantly upregulated. STRING protein–protein interaction network analysis placed *Mef2c* within a central regulatory hub (Figure 4C). To validate the sequencing data, we performed RT-qPCR on *Mef2c* and three randomly selected DEGs (*Cpne5*, *Emx2*, and *Doc2a*). The qPCR results were highly consistent with the RNA-seq FPKM values, confirming the reliability of our dataset (Figure 4D,E).

**Figure 5:**
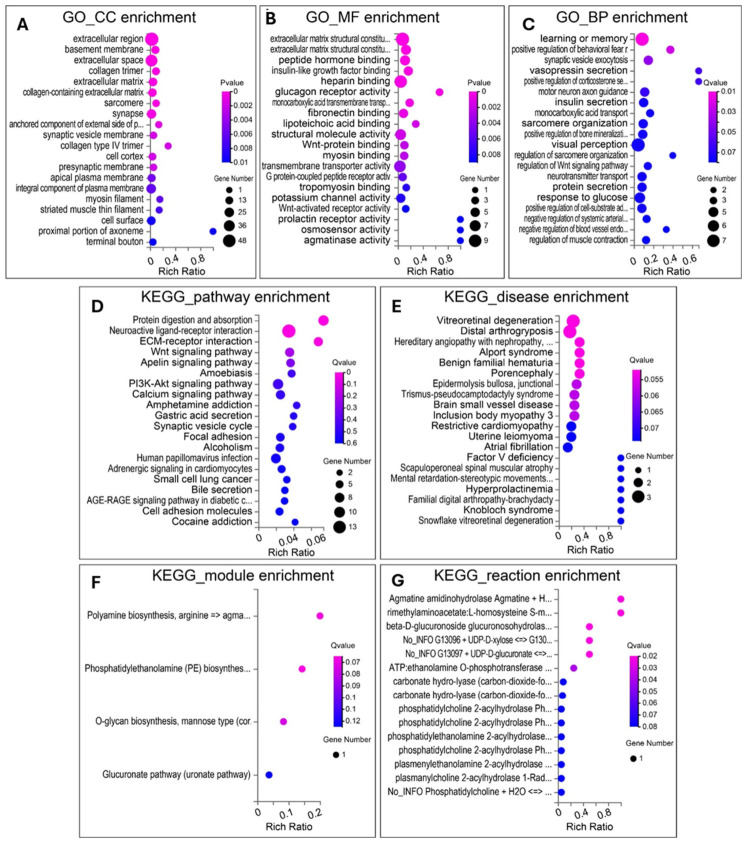
GO enrichment and KEGG enrichment analyses of DEGs. (**A**) GO-CC enrichment. (**B**) GO-MF enrichment. (**C**) GO-BP enrichment. (**D**) KEGG-pathway enrichment. (**E**) KEGG-disease enrichment. (**F**) KEGG-module enrichment. (**G**) KEGG-reaction enrichment.

Subsequent Gene Ontology (GO) and KEGG pathway enrichment analyses of all DEGs provided functional context. The DEGs were significantly enriched in GO biological processes related to learning/memory, regulation of fear response, synaptic neurotransmitter secretion, and axon guidance (Figure 5). KEGG analysis revealed enrichment in pathways including neuroactive ligand–receptor interaction, ECM–receptor interaction, and Wnt signalling.

This molecular profile, particularly the upregulation of *Mef2c* and the enrichment of pathways governing synaptic function, neuronal development, and behaviour, provides a plausible mechanistic basis for the observed deficits in hippocampal neurogenesis (NeuN/DCX phenotypes) and the emergence of depression-like behaviours following prenatal irradiation.

### 3.4. Prenatal Irradiation Upregulated MEF2C and EGR1 Protein Expression in the Hippocampal Dentate Gyrus

To validate and spatially localize the dysregulation of key transcription factors identified by RNA-seq, we performed immunohistochemical (IHC) staining and Western blot analysis on hippocampal tissue.

**Figure 6:**
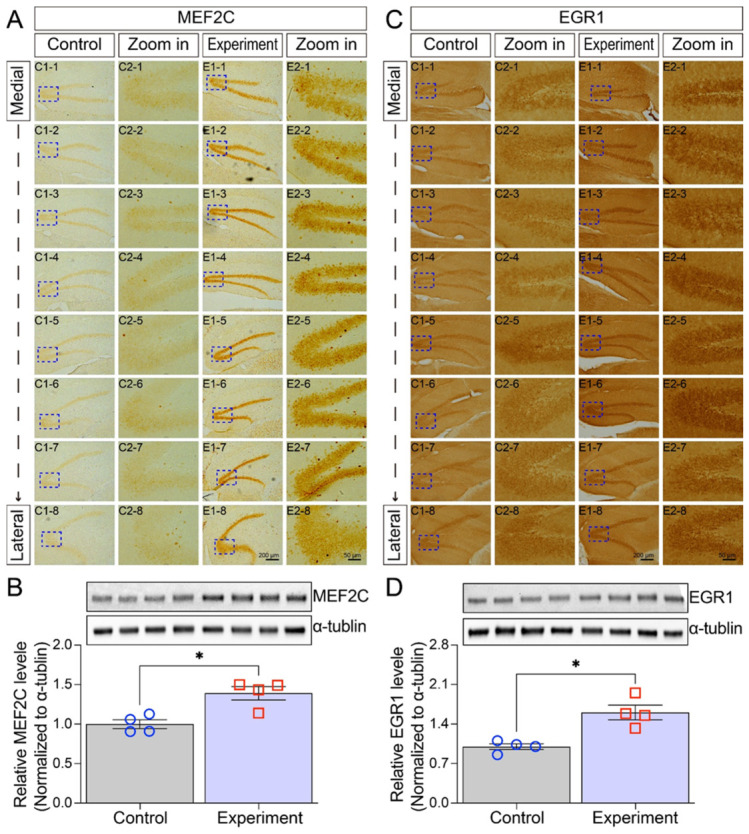
Irradiation significantly upregulates MEF2C and EGR1 protein levels in the hippocampus. (**A**) Immunohistochemical staining of MEF2C from medial to lateral hippocampal sections. MEF2C expression was predominantly localized to the granular layer of the dentate gyrus in irradiated mice. (**B**) Western blot analysis of MEF2C protein levels in the hippocampus of control and irradiated mice, with quantification performed using ImageJ software. (**C**) Immunohistochemical staining of EGR1. (**D**) Western blot analysis of EGR1 protein levels in the hippocampus of control and irradiated mice. Data are presented as mean ± SEM (*n* = 4 per group). * *p* < 0.05. Scale bar = 200 µm in E1-8 applies to C1-1 to C1-8, E1-1 to E1-7. Scale bar = 50 µm in E2-8 applies to C2-1 to C2-8, E2-1 to E2-7.

IHC revealed that both MEF2C and EGR1 proteins were prominently localized to the granular cell layer of the dentate gyrus. Critically, their expression levels were markedly elevated in irradiated mice compared to controls (Figure 6A,C).

This increase was confirmed at the total protein level by Western blot. Quantitative analysis demonstrated a significant upregulation of both MEF2C and EGR1 in hippocampal lysates from the irradiated group (Figure 6B,D).

### 3.5. miRSeq Identified miR-1843a-3p as a Putative Upstream Regulator of Mef2c

**Figure 7:**
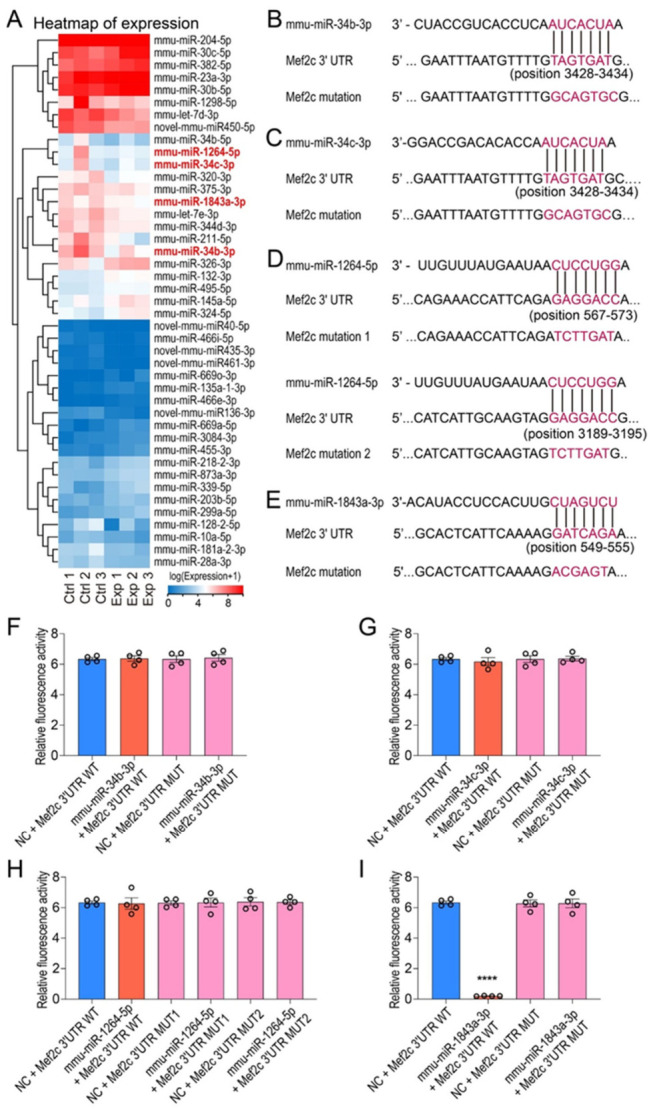
miRSeq analysis of hippocampal samples from control and irradiated mice, and luciferase reporter assay validation of direct miRNA–*Mef2c* interactions. (**A**) Heatmap showing 43 differentially expressed miRNAs between control and irradiated mice (fold change ≥ 1.5, *p* < 0.05). miRNAs predicted to target *Mef2c* mRNA are highlighted in red. (**B**–**E**) Sequence alignment of putative miRNA binding sites within the *Mef2c* 3′UTR, along with the corresponding mutant sequences. (**F**–**I**) Luciferase reporter assay activity. HEK293T cells were co-transfected with psiCHECK-2 constructs containing the *Mef2c* 3′UTR binding sites for each miRNA, together with miRNA mimics or scrambled control. Firefly luciferase and *Renilla* signals were measured 48 h post-transfection. Data are presented as mean ± SEM (*n* = 4 per group). **** *p* < 0.001.

To identify potential upstream regulators responsible for the observed MEF2C and EGR1 upregulation, we performed miRSeq on hippocampal samples. This analysis revealed 43 significantly dysregulated miRNAs (*p* < 0.05; fold-change > 1.5) in irradiated mice compared to controls (Figure 7A).

We next performed an in silico analysis to determine which of these miRNAs could directly target *Mef2c*. Using the TargetScan database to predict interactions with the 3′ UTR of *Mef2c*, we identified four candidate miRNAs: mmu-miR-34b-3p, mmu-miR-1264-5p, mmu-miR-1843a-3p, and mmu-miR-34c-3p (Table 3). The direct binding of these miRNAs to the *Mef2c* 3′ UTR was subsequently validated using a dual-luciferase reporter assay (Figure 7B–I).

A significant reduction in luciferase activity was observed in HEK293T cells co-transfected with the mmu-miR-1843a-3p mimic and a reporter construct containing the wild-type *Mef2c* 3′-UTR (position 549-555), compared to a scrambled mimic control (Figure 7E,I). This repression was abolished when the predicted binding site was mutated, confirming the specificity of the interaction. In contrast, luciferase activity was not significantly altered by the co-transfection of mimics for mmu-miR-34b-3p, mmu-miR-34c-3p, or mmu-miR-1264-5p (two predicted sites), despite the presence of predicted binding sites in the *Mef2c* 3′-UTR (Figure 7B–D,F–H).

These results demonstrate that mmu-miR-1843a-3p, but not the other candidate miRNAs, directly and specifically targets the 3′-UTR of *Mef2c* to regulate its expression.

## 4. Discussion

Ionizing radiation is a well-established environmental hazard with profound consequences for the developing central nervous system, particularly impacting neurogenesis and long-term neurological function. This study demonstrates that chronic prenatal gamma irradiation (5 Gy total dose) in C57BL/6 mice induces a specific adult phenotype characterized by reduced body weight, increased depression-like behavior, and significant impairment of hippocampal neurogenesis, without affecting anxiety, general exploration, or memory. While the cumulative dose of ~5 Gy used in this study is higher than typical environmental or occupational exposure levels, it is relevant to specific scenarios such as accidental radiation overexposure (e.g., nuclear accidents where pregnant women may receive high-dose-rate exposure before evacuation), or certain radiotherapeutic procedures involving the pelvic region during unrecognized early pregnancy. The dose-rate (12 mGy/h) and cumulative dose model our study employs are intended to simulate such acute, unfortunate exposure scenarios rather than background environmental radiation. Lower-dose studies (e.g., 100 mGy/day cumulative 1.8 Gy) have previously shown more subtle molecular changes without overt behavioral deficits, underscoring the dose-dependent nature of these effects [2]. Mechanistically, we link these outcomes to the dysregulation of a novel miR-1843a-3p/Mef2c/Egr1 pathway, providing a molecular framework for radiation-induced neurodevelopmental deficits.

Our findings contribute to a nuanced understanding of how exposure parameters dictate neurological outcomes. While acute neonatal high-dose-rate exposure (5 Gy) is known to cause severe neuropathology and depression [1], chronic postnatal low-dose-rate exposure (1.2 mGy/h to total dose 5 Gy) appears remarkably tolerated, showing no significant behavioural or neurogenic deficits [10]. The present study reveals that a prenatal, chronic high-dose-rate exposure (12 mGy/h to 5 Gy in total) occupies a distinct middle ground, eliciting specific depression-like behaviour and neurogenic disruption. This underscores the particular vulnerability of the prenatal period and suggests that dose-rate and developmental timing interact critically to determine functional sequelae. Furthermore, our observations align with studies showing that even lower total doses (~1.8 Gy) delivered at low dose-rates prenatally can induce sex-specific physiological changes without overt neuropsychiatric phenotypes [2], highlighting complex dose–response and sex-dependent relationships.

At the histological level, prenatal irradiation did not alter gross hippocampal architecture but specifically disrupted neurogenic processes. We observed a significant reduction in both DCX+ immature neurons and NeuN+ mature neurons within the dentate gyrus, accompanied by severe morphological aberrations in newborn neurons. This pattern of neurogenic impairment mirrors the acute effects seen after neonatal high-dose exposure [1] and contrasts with the resilience of neurogenesis to chronic low-dose-rate regimens [10]. This confirms that active neurogenesis during critical developmental windows is a primary target for high-dose-rate radiation injury.

Our integrated transcriptomic and biochemical analyses identified the transcription factor Mef2c as a central mediator of this effect. Mef2c is a master regulator of neuronal differentiation [11], synaptic plasticity [12], and activity-dependent gene programming [4,13], with human haploinsufficiency leading to severe neurodevelopmental disorders [14,15,16]. We found Mef2c mRNA and protein significantly upregulated in the hippocampus of irradiated mice. This upregulation was functionally linked to its downstream target Egr1, an immediate-early gene rapidly induced by cellular stress, including radiation [17,18]. Egr1 expression is tightly regulated by Mef2c [19]. The co-upregulation of MEF2C and EGR1 proteins provides a direct molecular link between the developmental insult and the disruption of transcriptional programs essential for neuronal maturation and circuit integration.

Crucially, we extended this pathway upstream by identifying mmu-miR-1843a-3p as a novel direct regulator of Mef2c. Luciferase assays confirmed its specific binding to the Mef2c 3′ UTR, and miRSeq revealed its significant downregulation following irradiation. While the broader role of this miRNA in neurogenesis remains to be fully elucidated, its identification here establishes a plausible regulatory mechanism: prenatal radiation exposure suppresses miR-1843a-3p, thereby de-repressing its target Mef2c. Elevated Mef2c levels then drive aberrant expression of downstream effectors like Egr1, ultimately disrupting the finely balanced gene networks required for normal neurogenesis and neuronal integration. An important caveat is that the observed upregulation of Mef2c could represent either a direct pathogenic contributor to the neurogenic deficits or a compensatory response aimed at restoring neuronal homeostasis following radiation injury. Distinguishing between these possibilities will require functional perturbation experiments, such as Mef2c knockdown or overexpression in the dentate gyrus of irradiated or control animals, respectively, combined with assessment of neurogenesis and behavior. Until such experiments are performed, the causal role of Mef2c upregulation in the observed phenotypes remains to be definitively established. We acknowledge that the transcriptomic analyses were performed with a limited sample size (*n* = 3 per group), which may limit the generalizability of the findings. However, the key differentially expressed genes, including Mef2c, were subsequently validated by qRT-PCR and Western blot in independent samples, supporting the robustness of these observations.

## 5. Conclusions

This study demonstrates that chronic prenatal gamma irradiation (12 mGy/h for 18 days, cumulative ~5 Gy) in C57BL/6 mice induces long-lasting and specific neurodevelopmental deficits that persist into adulthood. The adult offspring exhibited a distinct phenotype characterized by reduced body weight, selective increase in depression-like behaviors (increased immobility time in the tail suspension test), and significant impairment of adult hippocampal neurogenesis, while anxiety-like behaviors, general locomotor activity, and cognitive memory remained intact.

Mechanistically, the study identifies a novel regulatory axis, the miR-1843a-3p/*Mef2c/Egr1* pathway, as a key mediator of these radiation-induced deficits. Specifically, prenatal irradiation leads to the downregulation of mmu-miR-1843a-3p, which directly targets and represses Mef2c under normal conditions. The reduced miR-1843a-3p expression results in derepression and subsequent upregulation of MEF2C protein, accompanied by increased EGR1 expression. This dysregulation of activity-dependent transcriptional networks disrupts the finely balanced gene programs required for normal neuronal differentiation, synaptic plasticity, and circuit integration in the hippocampal dentate gyrus.

The findings highlight the particular vulnerability of the prenatal period to high-dose-rate radiation exposure, occupying a distinct outcome profile compared to acute neonatal exposure (severe pathology) or chronic postnatal low-dose-rate exposure (remarkable tolerance). The study conclusively links molecular dysregulation (miRNA and mRNA expression changes) to histological abnormalities (reduced and morphologically aberrant DCX^+^ newborn neurons, decreased NeuN^+^ mature neurons) and ultimately to behavioral dysfunction (depression-like phenotype).

**Figure 8:**
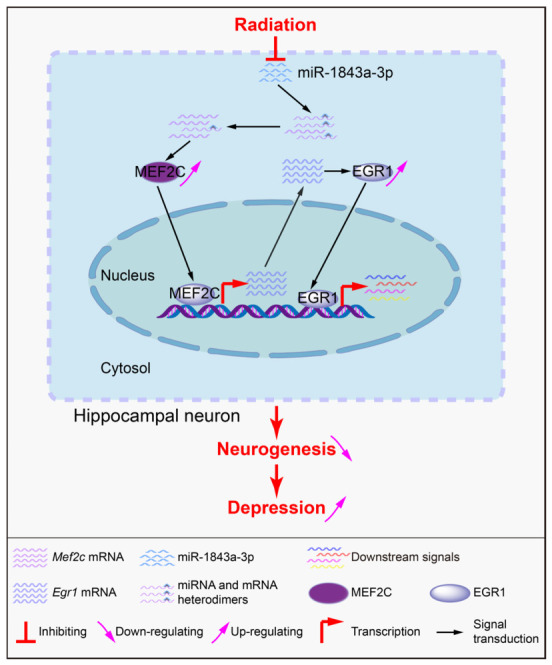
Proposed model of MEF2C signalling regulation. Prenatal irradiation induces differential expression of miRNAs targeting *Mef2c*, leading to altered MEF2C expression and downstream effects on neuronal morphology and function in the hippocampal dentate gyrus.

In conclusion, the miR-1843a-3p/*Mef2c/Egr1* axis represents a coherent mechanistic pathway through which prenatal gamma irradiation compromises adult brain function (Figure 8). These insights not only advance the understanding of radiation-induced neurodevelopmental disorders but also identify this pathway as a potential target for biomarker development and future neuroprotective interventions. It is important to note that only male offspring were examined in the present study. Given that previous work has demonstrated sex-specific physiological and molecular responses to prenatal low-dose-rate irradiation, future investigations incorporating female offspring are necessary to determine whether the miR-1843a-3p/*Mef2c/Egr1* axis is similarly dysregulated or contributes to distinct behavioral phenotypes in females. These findings advance our understanding of the molecular aetiology of radiation-induced neurodevelopmental deficits and identify the miR-1843a-3p/*Mef2c/Egr1* axis as a candidate pathway for future functional validation and potential biomarker development.

## 6. Limitations and Future Directions

Several limitations of this study should be acknowledged. First, as discussed above, the exclusive use of male offspring limits generalizability to females. Second, the relatively high cumulative dose (5 Gy) and high dose-rate (12 mGy/h) represent a specific exposure scenario that may not directly translate to lower-dose environmental exposures. Third, the transcriptomic analyses were performed with a small sample size (*n* = 3 per group for sequencing), although key findings were validated by qRT-PCR and Western blot in larger cohorts. Fourth, while we demonstrate a direct interaction between miR-1843a-3p and the Mef2c 3′UTR, the functional consequences of manipulating this interaction in vivo have not been tested.

Future studies should employ gain- and loss-of-function approaches to establish causality. For example, intracranial injection of miR-1843a-3p mimics or antagomirs into the dentate gyrus of irradiated or control mice, respectively, would directly test whether restoring or suppressing this miRNA is sufficient to rescue or recapitulate the neurogenic and behavioral phenotypes. Similarly, neuron-specific Mef2c knockdown using AAV-mediated CRISPR interference or overexpression using viral vectors could determine whether Mef2c upregulation is necessary and sufficient for the observed deficits. Finally, to explore the translational potential of this pathway, it would be valuable to examine whether circulating levels of miR-1843a-3p in blood or cerebrospinal fluid correlate with hippocampal expression and behavioral outcomes, potentially serving as a non-invasive biomarker for radiation-induced neurodevelopmental injury.

## Figures and Tables

**Table 1:** mRNA primer sequences for qRT-PCR.

Primer ID	Sequence
*Mef2c* Forward	GCACCAACAAGCTGTTCCAG
*Mef2c* Reverse	CAGATCTCCGCCCATCAGAC
*Cpne5* Forward	GGAGATCACGGTGTCATGCAG
*Cpne5* Reverse	TCCATCCAAAGACCACTTCCG
*Emx1* Forward	GGCGCTCAACTATCCTCACC
*Emx1* Reverse	GAAGCACCCAGGGGTAGAAG
*Doc2a* Forward	ATGAGGACAAGCTGAGCCAC
*Doc2a* Reverse	GTGGCCAGAGTGGAGAGTTC

**Table 2:** Primer sequences for luciferase reporter assay.

Primer ID	Sequence (5′-3′)	Notes
1	gactcatttagatcctcacac	reverse sequencing primers for psiCHECK2
2	cctccacttcagccaggagg	forward sequencing primers for psiCHECK2
3 *	aattctaggcgatcgctcgagTGTCACCTAACATCCAAGCA	forward primers for mouse *Mef2c* 3UTR with XhoI to psiCHECK2
4 *	attttattgcggccagcggccgcTCTGAAATTCTCATGTTAGT	reverse primers for mouse *Mef2c* 3UTR with NotI to psiCHECK2
5 **	cactcattcaaaagACGAGTaaaccattcagagaggaccatacctaccttaaaagaa	mmu-miR-1843a-3p mutation to ACGAGT
6 **	atggtcctctctgaatggtttACTCGTcttttgaatgagtgccatacgccaatgatatg	
7 **	gaaaccattcagaTCTTGATatacctaccttaaaagaaaagagaagaaaggaaagg	mmu-miR-1264-5p S1 mutation to TCTTGAT
8 **	cttttaaggtaggtatATCAAGAtctgaatggtttctgatccttttgaatgagtg	
9 **	gtcatcattgcaagtagTCTTGATgtaaatggcattttacatgactgcaagtattg	mmu-miR-1264-5p S2 mutation to TCTTGAT
10 **	aaaatgccatttacATCAAGActacttgcaatgatgactgttgtctaatatgctac	
11 **	atttaatgttttgGCAGTGCgaatatgaaaatgcctgtcatctttagatcattga	mmu-miR-34b/c-3p mutation to GCAGTGC
12 **	attttcatattcGCACTGCcaaaacattaaattccacctttgggaaaaaaataagc	

Note: * Sequences marked in red in primers 3 & 4, restriction enzyme sites. ** Sequences marked in red in primer 5–12, mutation sites.

**Table 3:** Differentially expressed miRNAs targeting *Mef2c*.

MicroRNA Symbol	Expression								log2(Exp/Ctrl)	Pvalue (Exp/Ctrl)
	Ctrl 1	Ctrl 2	Ctrl 3	Exp 1	Exp 2	Exp 3	Ctrl Average	Exp Average		
mmu-miR-34b-3p	96.56	293.586	78.132	23.84	64.28	33.01	156.092	40.377	−1.523319549	1.43 × 10^−4^
mmu-miR-1264-5p	20.846	139.152	20.88	18.804	24.744	21.02	60.293	21.523	−1.02719965	0.018144898
mmu-miR-1843a-3p	60.268	35.628	65.178	36.407	35.884	28.322	53.691	33.538	−0.642570783	0.029282449
mmu-miR-34c-3p	18.928	96.995	17.393	11.92	27.575	17.862	44.439	19.119	−0.889305871	0.037617535

## Data Availability

The original contributions presented in this study are included in the article. Further inquiries can be directed to the corresponding authors.
